# Editorial: Adenoviral Infection and Immunity, and Adenoviral Vectors for Gene Therapy Applications

**DOI:** 10.3389/fmicb.2022.869059

**Published:** 2022-03-24

**Authors:** Qiwei Zhang, Yiqiang Li, Pascal Fender, Bin Yu, Liqiang Feng, Ling Chen, Xianghui Yu

**Affiliations:** ^1^Guangdong Provincial Key Laboratory of Virology, Institute of Medical Microbiology, Jinan University, Guangzhou, China; ^2^Guangdong Provincial Key Laboratory of Tropical Disease Research, School of Public Health, Southern Medical University, Guangzhou, China; ^3^Institut de Biologie Structurale (IBS), Université Grenoble Alpes, CNRS, CEA, Grenoble, France; ^4^National Engineering Laboratory for AIDS Vaccine, School of Life Sciences, Jilin University, Changchun, China; ^5^Guangdong Laboratory of Computational Biomedicine, Guangzhou Institutes of Biomedicine and Health (GIBH), Chinese Academy of Sciences, Guangzhou, China; ^6^State Key Laboratories of Respiratory Diseases, The First Affiliated Hospital of Guangzhou Medical University, Guangzhou, China; ^7^Key Laboratory for Molecular Enzymology and Engineering of the Ministry of Education, School of Life Sciences, Jilin University, Changchun, China

**Keywords:** adenovirus, infection, immunity, adenoviral vector, gene therapy

Human adenoviruses (HAdVs) are associated with gastroenteritis, conjunctivitis, respiratory and urinary tract infections, which generally have low clinical relevance. However, they can cause serious morbidity in immunocompromised patients. In addition, some mutant or intertypic recombinant types can also cause outbreaks of community-acquired pneumonia (CAP) (Walter, 2020). There is no efficient treatment for HAdV infections approved so far. Remarkably, adenoviruses are still the most widely used vectors in the clinical gene therapy field (https://a873679.fmphost.com/fmi/webd/GTCT), with applications ranging from oncolytic therapies to vaccinations. Therefore, exploring the viral infection and vector-induced host immune mechanism and engineering more ideal HAdV vectors for the treatment of tumors and other diseases are important in basic research and biotechnologies in microbiology.

Although HAdVs have been found for almost 70 years and many of their basic properties have been discovered, some important molecular mechanisms such as the infection and innate immunity in epithelial cells, immune cells, and hematopoietic stem cells still need to be entirely characterized. To date, interactions between HAdVs and host factors and the antiviral immunity *in vivo* have been investigated preliminarily (Chéneau et al.; Tran et al., 2021; Eichholz et al., 2022), which still needs further investigation. HAdV vector-based gene therapy shows broad application potentials. However, nanomedicine based on HAdV vectors needs to consider the effects of dosage and potential toxicity, which limits their use in clinical trials (Qu et al., 2019). Many groups are working on modifying the natural properties of HAdVs to turn them into better tools for gene transfer, oncolytic virotherapy, or vaccines (Yan et al., 2021; Zhang et al., 2021a). Increased knowledge of HAdV biology, especially mechanisms in its infection and immunity, will help develop novel strategies to improve the therapeutic activities to promote the development of HAdV research in infection, immunity, and gene therapy. This Research Topic established a platform for exchanging of the latest HAdV-associated information. We, rigorous peer review, processed 12 manuscripts in total, of which 4 were rejected while 8 were published.

HAdV-55 is an important pathogen causing CAP, of which the receptor was identified as Desmoglein 2 (DSG2) (Zhang et al., 2021b). Chen et al. studied the epidemic status of HAdV-55 in Guangzhou, southern China, from May 2017 to April 2019 and found that HAdV-55-positive cases increased significantly. The whole-gene sequencing (WGS) alignment of the strain isolated from a case of death revealed that it has a high degree of homology with the prototype strain in China 2006, while the other three isolated from Shanxi, northern China, are similar to another strain isolated earlier in the same area. The four HAdV-55 strains are divided into subtype b according to the absence of the insertion of “CCATATCCGTGTT”. This study also confirmed an earlier report that HAdV-55 had highly conserved capsid proteins and genomes (Cheng et al.).

In addition to causing fatal pneumonia, HAdVs can also cause various acute respiratory infections(ARIs). Wen et al. took multiplex PCR and capillary electrophoresis, hypervariable region of the hexon gene as the target, to identify HAdV types in hospitalized children in Wenzhou, southeastern China. They analyzed the correlation between HAdV types and the symptoms of different ARIs and found that HAdV-3,−7,−2, and−1 were the main types of ARIs in hospitalized children in this area. Among children with pneumonia, tonsillitis, bronchitis, and upper respiratory tract infection, the HAdV types are significantly different. Furthermore, they showed that HAdV-7 caused more severe pneumonia in children than HAdV-3.

HAdV-3, as one of the most common types circulating in Chinese children and causing ARIs, has received much attention. Duan et al. analyzed the main capsid proteins (hexon, penton, fiber) and early genes (E1, E2, E3, and E4) through the WGS of HAdV-3 obtained in 6 cities in China from 2014 to 2018. All the strains were found to have a high degree of homology with the Chinese reference strains, while the HAdV-3 prototype strain was in a separate cluster, and amino acid substitutions and insertions were found in the penton region of which most have not been reported so far. Thus its specific significance needs to be studied deeply. Moreover, for HAdV-3, through recombination analysis, it was found that IIIa precursor, penton bases, and partial regions of protein VII precursor gene were all derived from HAdV-7.

In clinical practice, HAdV-mediated complications contribute to serious morbidity and mortality in immunocompromised patients, especially in the pediatric hematopoietic allogeneic stem cell transplant (HSCT) setting (Kosulin et al.). By sequence alignment, through WGS, combined with epidemiological data, Myers et al. found that there was a small-scale outbreak within HSCT inpatients with HAdV-related deaths as the source of infection. Those patients had mixed infections frequently, which are prone to induce recombinant mutations and superinfections. Therefore, Myers et al. proposed that HAdV-WGS should be included in routine clinical monitoring, especially for immunosuppressed patients with high HAdV positivity.

Chen et al.; Wen et al.; Duan et al.; Myers et al. have reported that HAdVs can endanger lives seriously in special populations. The epidemic trend and recombination of HAdVs can be understood furtherly by the WGS method. Zhao et al. provided a fast and accurate method to obtain the WGS of HAdVs, which is based on the high-precision first-generation sequencing technology without PCR amplification. Zhao et al. directly used a set of walking primers to the sequence. The whole- genomic DNA is taken as template instead of PCR amplicons, which is a fast and accurate method for acquiring the WGS of common HAdV species B, C, and E. More attractive is that this method can be applied to the WGS of other DNA viruses.

This Research Topic has also deeply discussed the HAdV-associated animal vaccine model and immunology. Gokumakulapalle et al. used HAdV-11 prototype strain (HAdV11p) encoding green fluorescent protein (GFP) to evaluate its tropism and replication in the canine (MDCK), hamster (CHO), and mouse (McCoy and C127) cell lines. They found that HAdV11p can infect the three animal cell models, in which the expression of GFP in MDCK is relatively high. Nevertheless, the effective release of the virus after the infection has not been observed in these cell lines. They provided an HAdV vector with the replication ability to develop an oral SARS-CoV-2 vaccine.

Wang et al. analyzed the immunological features of ancestral loop 1 and 2 regions of HAdV sequences by sequence alignment and homology modeling methods. They found that the tower region of the hexon protein had a high degree of variability, while the neck and base area remained constant among different types, thus successfully predicting the common ancestral sequence of the HAdV hexon. Healthy adults rarely harbored neutralizing antibodies against the epitopes on the consensus ancestor of adenoviral hexon, which is of great significance for researching HAdV vaccine vectors.

An excellent review was included in this Research Topic, discussing the prospects of oncolytic adenovirus (OAds) in tumor immunotherapy. Zhao et al. described two fields: one is OAds genome modification strategies, such as capsid modification and small deletion of pivotal virus genes. The other is the OAds application in tumor immunotherapy, such as OAds combined with anti-programmed death 1/programmed death-ligand 1, which significantly affects anti-tumor therapy. Zhao et al. also proposed that the current immunotherapy still faced severe challenges such as antiviral immune response and tumor microenvironment barriers.

This Research Topic contains a total of 8 papers on HAdV-related infections, immunity, and HAdV vectors for gene therapy. We hope these papers contribute to research teams, clinicians, and students interested in HAdVs. Finally, we sincerely thank all authors and reviewers for their valuable contributions to this Research Topic.

## Author Contributions

QZ and YL drafted the editorial while PF, BY, LF, LC, and XY contributed to editing. All authors contributed to the article and approved the submitted version.

## Funding

This work was supported by grants from the National Key Research and Development Program of China (2018YFE0204503), National Natural Science Foundation of China (32170139), and Natural Science Foundation of Guangdong Province (2018B030312010 and 2021A1515010788).

## Conflict of Interest

The authors declare that the research was conducted in the absence of any commercial or financial relationships that could be construed as a potential conflict of interest.

## Publisher's Note

All claims expressed in this article are solely those of the authors and do not necessarily represent those of their affiliated organizations, or those of the publisher, the editors and the reviewers. Any product that may be evaluated in this article, or claim that may be made by its manufacturer, is not guaranteed or endorsed by the publisher.
